# Disruption of CDK7 signaling leads to catastrophic chromosomal instability coupled with a loss of condensin-mediated chromatin compaction

**DOI:** 10.1016/j.jbc.2023.104834

**Published:** 2023-05-17

**Authors:** Katrina M. Piemonte, Bryan M. Webb, Jessica R. Bobbitt, Parth R. Majmudar, Leslie Cuellar-Vite, Benjamin L. Bryson, Nicholas C. Latina, Darcie D. Seachrist, Ruth A. Keri

**Affiliations:** 1 Department of Cancer Biology, Lerner Research Institute, Cleveland Clinic, Cleveland, Ohio, USA; 2 Department of Pharmacology, Case Western Reserve University School of Medicine, Cleveland, Ohio, USA; 3 Department of Pathology, Case Western Reserve University School of Medicine, Cleveland, Ohio, USA; 4 Department of Genetics and Genome Sciences, Case Western Reserve University School of Medicine, Cleveland, Ohio, USA; 5 Department of General Medical Sciences-Oncology, Case Western Reserve University School of Medicine, Cleveland, Ohio, USA

**Keywords:** chromosomal instability, CDK7, condensin, CT7001, cyclin dependent kinase 7, mitosis, mitotic catastrophe, SMC2, THZ1, TNBC, triple negative breast cancer

## Abstract

Chromatin organization is highly dynamic and modulates DNA replication, transcription, and chromosome segregation. Condensin is essential for chromosome assembly during mitosis and meiosis, as well as maintenance of chromosome structure during interphase. While it is well established that sustained condensin expression is necessary to ensure chromosome stability, the mechanisms that control its expression are not yet known. Herein, we report that disruption of cyclin-dependent kinase 7 (CDK7), the core catalytic subunit of CDK-activating kinase, leads to reduced transcription of several condensin subunits, including structural maintenance of chromosomes 2 (*SMC2*). Live and static microscopy revealed that inhibiting CDK7 signaling prolongs mitosis and induces chromatin bridge formation, DNA double-strand breaks, and abnormal nuclear features, all of which are indicative of mitotic catastrophe and chromosome instability. Affirming the importance of condensin regulation by CDK7, genetic suppression of the expression of SMC2, a core subunit of this complex, phenocopies CDK7 inhibition. Moreover, analysis of genome-wide chromatin conformation using Hi-C revealed that sustained activity of CDK7 is necessary to maintain chromatin sublooping, a function that is ascribed to condensin. Notably, the regulation of condensin subunit gene expression is independent of superenhancers. Together, these studies reveal a new role for CDK7 in sustaining chromatin configuration by ensuring the expression of condensin genes, including SMC2.

Cyclin-dependent kinases (CDKs) are a family of serine/threonine phosphotransferases activated by cyclins. The CDKs are broadly divided into proteins that regulate cell cycle progression, those that control transcription, and those that are considered atypical, which remain poorly characterized (1). Uniquely, CDK7 fits into two of the three categories and is able to regulate cell cycle progression and transcription (2). CDK7 is the catalytic component of CDK-activating kinase (CAK), a heterotrimeric complex that also includes cyclin H and ménage-à-trois 1 (MAT1) (3). CAK governs cell cycle progression through T-loop phosphorylation of CDKs 1, 2, 4, and 6. As a member of the transcription factor IIH complex, CAK also controls transcription initiation and elongation through direct phosphorylation of serine 5 and 7 in the C-terminal domain of RNA polymerase II (RNA Pol II) and indirectly by activating CDK9, which phosphorylates RNA Pol II at serine 2. In addition to its modulation of RNA Pol II, CAK also regulates transcription by phosphorylating several transcription factors (3).

Given its role in the cell cycle and transcription, CDK7 is a promising therapeutic target in cancer (4, 5, 6, 7). CDK7 inhibitors (CDK7is) have significant efficacy in various preclinical cancer models with limited systemic toxicity (8, 9, 10, 11, 12). Based on these results, at least five CDK7i (SY5609, SY-1365-terminated, XL02, CT7001/ICE0942, Q901) are under investigation in phase I/II clinical trials (3, 12, 13, 14, 15). While promising, achieving maximal utility of such inhibitors will require complete elucidation of the mechanisms by which CDK7 controls cell growth and viability to permit prospective selection of tumors that would be highly sensitive to CDK7i, predict off-target effects, identify mechanisms of drug resistance, and develop optimal therapeutic combinations.

In addition to directly regulating the cell cycle and transcription initiation/elongation, CDK7 is a key regulator of superenhancers (SEs) (8, 16). These are high-density clusters of enhancers occupied by master transcription factors that promote cell identity and ensure the expression of oncogenes when co-opted in cancer (17, 18). It has been suggested that targeting SEs will broadly block transcriptional addiction of cancer cells, and several small-molecule inhibitors have been developed to suppress the activity of various proteins at SEs (19), including CDK7. In some studies, the efficacy of CDK7i has been attributed to the disassembly of SEs that drive expression of specific oncogenic transcription factors (20, 21, 22, 23). In T-cell acute lymphoblastic leukemia, THZ1, a covalent CDK7i, profoundly suppresses the SE that drives expression of *RUNX1,* an oncogenic transcription factor in this disease (8). Likewise, THZ1 suppresses *MYCN* gene expression in neuroblastoma, consequently disrupting the MYCN-regulated transcriptome (20). In other cases, it has been suggested that global suppression of SEs extending beyond individual transcription factor genes underlies CDK7i efficacy. For example, in triple negative breast cancer (TNBC) cells, use of THZ1 revealed a set of genes deemed to be an “Achilles Cluster,” *i.e.*, genes to which TNBC cells are addicted (16). These genes include components of protumorigenic signaling pathways as well as transcription factors.

While numerous studies support SE disruption as the primary mechanism of action that leads to cell death when CDK7 activity is blocked, others have suggested that CDK7 is necessary to maintain genome integrity. Wong and colleagues reported that a novel CDK7 inhibitor (YKL-5-124) did not disrupt SEs but instead specifically impacted the cell cycle function of CDK7 (24). This agent induced profound replicative stress, DNA damage, and micronucleation (24), an indicator of chromosomal instability (CIN) in models of small cell lung cancer (SCLC). The ability of CDK7i to induce CIN was further demonstrated in several models of hepatocellular carcinoma (HCC) using THZ1. In this case, CIN was proposed to result from disruption of MYC-driven cell cycle progression (25).Given the myriad of activities associated with CDK7, the mechanisms by which it prevents CIN remains unknown. Specifically, it is unclear whether the CIN that occurs in response to CDK7i is dependent upon changes in the transcription of core genes involved in DNA damage and genomic instability or if it is due to the disruption of key events during cell cycle progression. Moreover, it is not currently known if the genome instability induced by CDK7i is broadly applicable to cancers of multiple lineages or if this effect is restricted to SCLC or cancers that are dependent on MYC, such as HCC.

We addressed these questions by assessing the impact of inhibiting CDK7 on growth and genome stability in models of TNBC, an aggressive, genomically heterogeneous disease with high rates of aneuploidy and CIN (26, 27, 28, 29, 30). *CDK7* expression has also recently been reported to be a candidate biomarker for poor prognosis in breast cancer (31). While previous reports have suggested that CDK7i may be effective in TNBC due to disruption of SEs, we asked whether CDK7 may be essential for maintaining genome stability, and the potential mechanisms involved. We report that genomic and pharmacologic suppression of CDK7 leads to several characteristics of mitotic catastrophe and excessive CIN. In addition, loss of CDK7 activity induces chromosome bridges and DNA double-strand breaks, indicating that CDK7 is globally necessary to sustain genome integrity.

Mechanistically, we discovered that CDK7 is essential for sustained gene expression of members of the condensin complex. This complex is responsible for compacting chromatin and is essential for preparing chromosomes for proper segregation during mitosis and maintenance of genome integrity throughout this process (32). Chromosomes that are formed following condensin loss are described as “disheveled” (33), “swollen,” “cloud-like,” and “with a fuzzy appearance” (34, 35). Chromosomes with these morphologies often result in binucleated cells, lagging chromatin at anaphase, and subsequent CIN (33), similar to the impact of CDK7 repression reported here. Supporting the importance of CDK7 in regulating condensins, direct silencing of a core subunit, structural maintenance of chromosomes 2 (SMC2), phenocopied the effects of CDK7i. The impact of CDK7 on condensin gene expression appears to be independent of SEs, as these genes lack such elements. Given the key function of the condensin complex in driving three-dimensional (3D) genome organization, we postulated that, by modulating condensin, CDK7 is essential for protecting chromosomal DNA from damage by promoting its condensation (36, 37). High-throughput chromosome conformation capture (Hi-C) analysis confirmed that THZ1 disrupted 3D chromatin architecture, with significant loss of chromatin sublooping and increased long-range interactions and interchromosomal interactions. Together, these data reveal a previously undescribed role of CDK7 that ensures proper expression of condensin subunits and reveals a mechanism by which CDK7 sustains genome integrity by ensuring proper compaction and protection of chromosomal DNA.

## Results

### CDK7 is necessary for normal mitoses in TNBC cells

Prior reports have indicated that TNBC cells are sensitive to CDK7i, such as THZ1 and CT7001 (16). We confirmed the reliance on CDK7 activity for the models used in the current study (MDA-MB-231, HCC38, and MDA-MB-468) using a small-molecule inhibitor, THZ1. THZ1 decreased the growth of all three cell lines after 72 h with IC_50_ values in the “low-dose” range (38) of 25 to 75 nM (Fig. S1*A*). Similar results were observed with CT7001, which is also currently being evaluated in clinical trials (8, 9, 10, 11, 12) (Fig. S1*B*). As expected (16), growth suppression was accompanied by loss of RNA Pol II activity due to loss of total RNA Pol II and no compensatory upregulation of phosphorylated RNA Pol II was observed (Fig. S1, *C* and *D*), confirming the ability of THZ1 inhibitors to repress transcription in these cells, even at low doses. Transient blockade of CDK7 activity also suppressed colony formation when cells were treated with THZ1 for only 72 h and then replated in drug-free medium for several days (Fig. S1, *E* and *F*). Thus, short-term disruption of CDK7 activity leads to irreversible growth suppression, indicating that sustained CDK7 signaling is necessary to prevent catastrophic events that permanently block cell growth.

**Figure 1:**
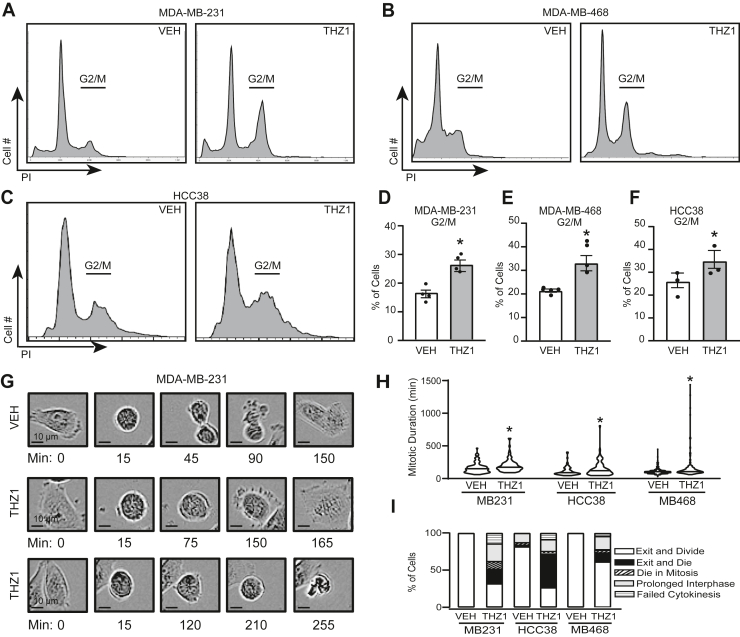
**CDK7 is necessary for normal mitoses in triple negative breast cancer cells.** Propidium iodide flow cytometry analysis was used to delineate changes in cell cycle phases with *A*, MDA-MB-231, *B*, MDA-MB-468, or *C*, HCC38 cells treated with THZ1 (75 nM, 50 nM, 50 nM, respectively) compared with vehicle (DMSO) after 72 h. *D*–*F*, quantitation of significantly changed G2/M populations. Means are ± SEM. *G*, live cell images were captured with an Incucyte microscope of MDA-MB-231, HCC38, and MDA-MB-468 following disruption of CDK7 activity by THZ1 treatment. Representative images are shown of MDA-MB-231 cells with indicated time points following identification of cells entering mitosis. *Top*, normal mitosis; *middle*, failed cytokinesis; *bottom*, die in mitosis. *H*, quantitation of mitosis duration of at least 60 cells per treatment per cell line. *Horizontal solid lines* are means. *I*, quantitation of mitotic fates (≥60 cells) treated with vehicle (DMSO) or THZ1. ∗ = *p* < 0.05.

Given the established roles of CDK7 in promoting cell cycle progression and its reported impact on chromosomal stability (24, 25), we questioned whether loss of CDK7 activity may induce mitotic catastrophe. Mitotic catastrophe is an oncosuppressive mechanism (39, 40). This type of cell death or senescence occurs either during or immediately following mitosis in response to gross failures in mitotic progression and DNA damage (39). To determine if the irreversible growth inhibition that occurs with disruption of CDK7 activity is due to mitotic catastrophe, we first assessed whether CDK7i hinders cell cycle progression, particularly in mitosis, and found that treatment with THZ1 for 72 h (50–75 nM) caused an arrest in G_2_/M (Fig. 1, *A*–*F*). These data are supported by a previous study demonstrating a significant G_2_/M arrest in *MYC-*amplified neuroblastoma cells that becomes more pronounced from 24 h to 48 h (20). Interestingly, short-term (24 h) THZ1 treatment in the TNBC cell line, MDA-MB-468, did not result in significant G_2_/M arrest (16). These studies, in conjunction with the data herein, indicate that the time frame of treatment is essential for observing the mitotic consequences of CDK7 inhibition. Specifically, cells treated for a short time course are still able to progress through early mitoses following CDK7i, while at later time points, there is significant mitotic delay due to the accumulation of irreversible errors. Using live-cell imaging, we found that mitotic progression is delayed when CDK7 is disrupted with a small molecule inhibitor (THZ1) (41, 42, 43). For each cell line, we quantified the duration of mitosis as well as the postmitotic fates of daughter cells in vehicle and CDK7i-treated cells (for detailed description of fate identification, see Methods, Live Cell Imaging, Incucyte). All three cell lines exhibited a significant increase in the average time spent in mitosis following 72 h of THZ1 exposure (Fig. 1, *G*–*I*). We also observed profound changes in mitotic cell fate (Fig. 1*I*). Compared with vehicle, blocking CDK7 activity substantially reduced the percentage of cells that could exit mitosis and engage in a second round of replication and division (exit and divide). This was complemented by an increase in the percentage of cells that died during mitosis (example shown in Fig. 1*G*, bottom row), exited mitosis and then died, underwent a prolonged interphase (>25 h), or failed to complete cytokinesis (example shown in Fig. 1*G*, middle row). Although the specific fraction of outcomes varied across cell lines, each line displayed defects in mitotic progression that are collectively indicative of mitotic catastrophe following the disruption of CDK7 function.

### CDK7 activity maintains chromosomal stability

**Figure 2:**
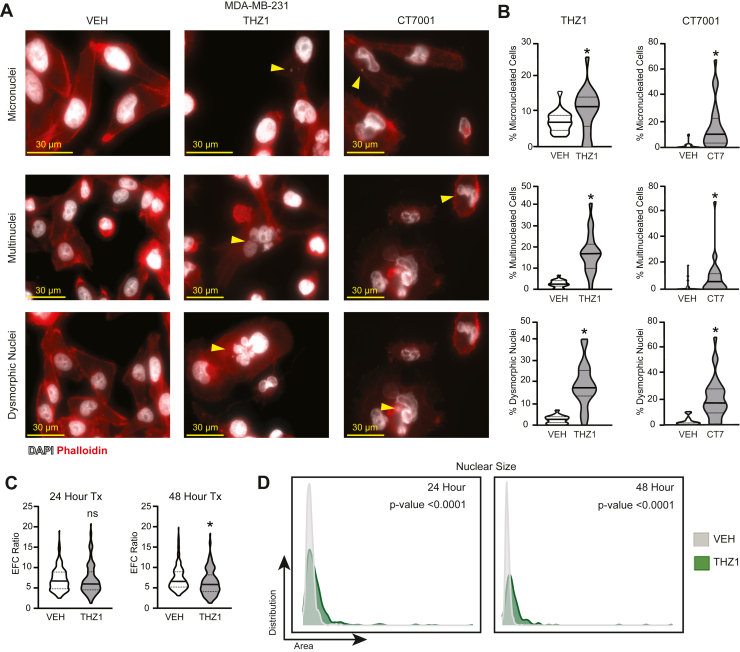
**CDK7 maintains chromosome stability.***A*, MDA-MB-231 cells were treated with vehicle (DMSO), THZ1 (75 nM), or CT7001 (CT7, 750 nM) for 72 h. Cells were stained with DAPI (nuclei, *white*) and phalloidin (actin, *red*). Micronuclei, dysmorphic nuclei, and multiple nuclei are indicated by *arrowheads*. Micrograph representing multinucleated cell and dysmorphic nuclei in MDA-MB-231 cells treated with CT7001 is reused. This micrograph contains both phenotypes as indicated by the *arrows*. *B*, quantitation of the percentage of cells from A with each phenotype is shown. *Horizontal solid lines* are mean, *dashed lines* are quartiles. At least 60 cells were counted per treatment per cell line. *C*, elliptical Fourier coefficient (EFC) ratios of MDA-MB-231 nuclei following 24- and 48-h treatment with THZ1. Lower values indicate greater dysmorphia. *D*, nuclear size in MDA-MB-231 cells following 24- and 48-h treatment with THZ1. Three biological replicates were completed per experiment. Wilcoxon signed-rank test was used to assess statistical significance of nuclear size changes. ∗ = *p* < 0.05, ns = not significant.

**Figure 3:**
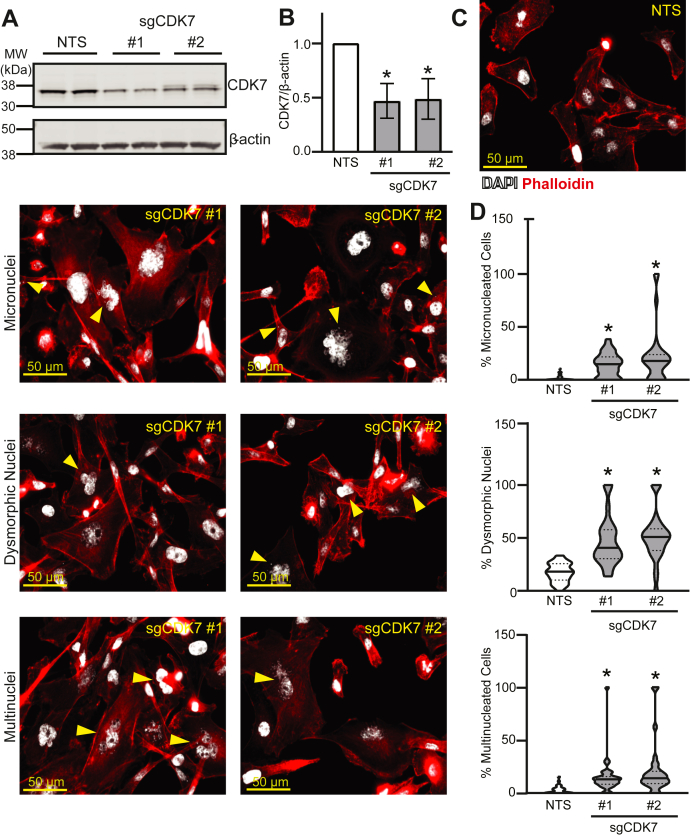
**Genetic disruption of *CDK7* phenocopies pharmacologic inhibition of CDK7.***A*, MDA-MB-231 Cas9-expressing cells were transfected with nonspecific sgRNA (NTS) or sgRNA targeted to *CDK7* (sgCDK7 #1, sgCDK7 #2). Representative Western blot demonstrating CDK7 protein loss. *B*, quantitation of CDK7 knockout in MDA-MB-231 cells relative to β-actin. Bars are means ± SEM. *C*, to visualize nuclear atypia, 72 h after sgRNA transfection, MDA-MB-231 Cas9 cells were stained with DAPI (*white*) and phalloidin (*red*). Micronuclei, dysmorphic nuclei, and multiple nuclei are indicated by *arrowheads*. *D*, quantitation of each phenotype is shown. Horizontal solid lines are means; *dashed lines* are quartiles. At least three biological replicates were completed per experiment in duplicate. ∗ = *p* < 0.05.

As a consequence of mitotic defects, cells often display changes in nuclear morphology including micronucleation, multinucleation, dysmorphic nuclei, increased nuclear size, and loss in circularity (44, 45, 46, 47, 48), all of which are hallmarks of chromosome instability or CIN. To determine if the mitotic defects that occur in response to CDK7 signaling induce CIN, MDA-MB-231 and HCC38 cells were treated with CDK7i (THZ1) or vehicle and the percentage of cells with micro-, multiple, and dysmorphic nuclei were quantified. CDK7 blockade significantly increased all three phenotypes (Figs. 2, *A* and *B* and S2, *A* and *B*). In addition, dysmorphic nuclei were independently quantified using elliptical Fourier analysis to assess nuclear contour irregularities, and this confirmed an accumulation of abnormal nuclear shapes following the inhibition of CDK7 activity (Figs. 2*C* and S2*C*). The elliptical Fourier coefficient (EFC) compares the sum of the sizes of elliptical Fourier harmonics 2 to 15 to the size of the first harmonic. Compared with the average of control (vehicle) nuclei, treated (THZ1) nuclei had lower EFC values, indicating more dysmorphic nuclei. Similar results were observed with a second CDK7i, CT7001 (Fig. 2, *A* and *B*). The phenotypes observed following CDK7i were recapitulated in Cas9-expressing MDA-MB-231 cells following transfection with *CDK7* targeted sgRNA (Fig. 3, *A*–*D*). Together, these data indicate that the nuclear features observed with CDK7 inhibition are not due to off-target effects of the inhibitors on CDK12/13 or other kinases. Lastly, disrupting CDK7 also resulted in increased nuclear size (Figs. 2*D* and S2*D*). Such increases have been associated with either increased genetic material or alterations in chromosome architecture (48, 49, 50). Together, these results suggest that sustained activity of CDK7 may be necessary to sustain genome stability.

**Figure 4:**
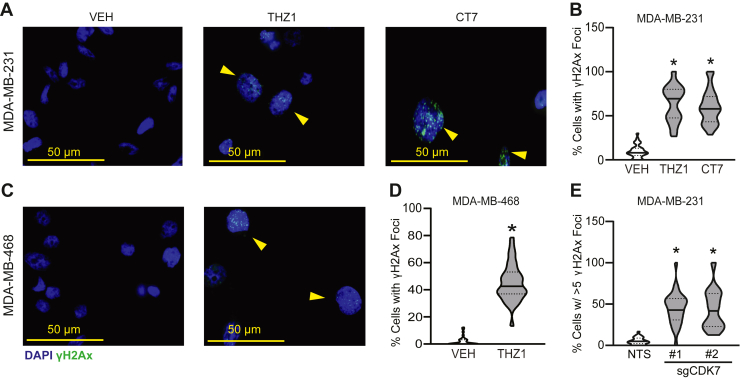
**CDK7 inhibition induces DNA double-strand breaks.***A*, MDA-MB-231 *C*, MDA-MB-468 cells were treated with vehicle (DMSO), THZ1 (75 nM), or CT7001 (CT7, 750 nM) and MDA-MB-468 cells were treated with vehicle or THZ1 (50 nM) for 48 h. Cells were then immunostained for γH2AX (*green*) and counterstained with DAPI (*blue*). *Yellow arrowheads* indicate nuclei with γH2AX foci. *B* and *D*, quantitation of the percentage of cells with γH2AX foci in MDA-MB-231 and MDA-MB-468, respectively. *E*, MDA-MB-231 Cas9-expressing cells were transfected with nonspecific sgRNA (NTS) or sgRNA targeted to CDK7 (sgCDK7 #1, sgCDK7 #2). Cells were stained as *A* and quantified as described above. *Horizontal solid lines* are means; *dashed lines* are quartiles. ∗ = *p* < 0.05.

**Figure 5:**
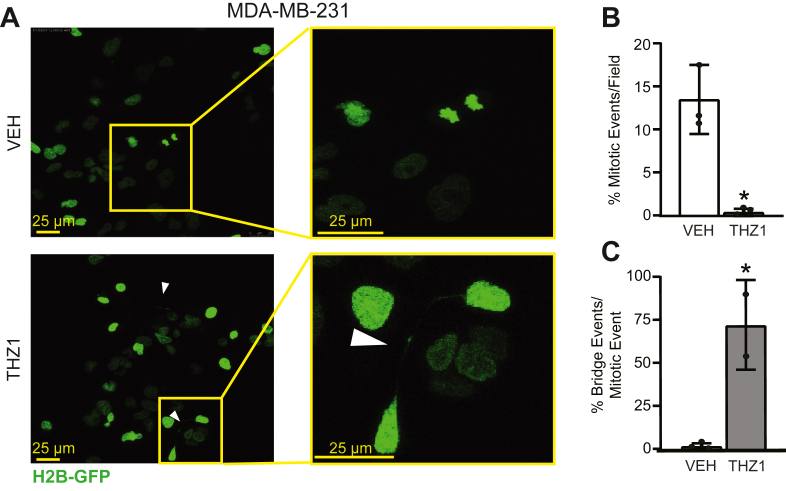
**CDK7 inhibition is associated with chromosome bridge formation during late (>30 h) mitotic events.***A*, live-cell confocal imaging of MDA-MB-231 cells stably transfected with an H2B-GFP expression vector were treated with vehicle or 75 nM THZ1 for 50 h. Cells were imaged from 30 to 50 h post initiation of treatment. *White arrowhead* indicates a chromatin bridge between two cells. *B*, quantitation of mitotic events after CDK7 inhibition. *C*, quantitation of chromatin bridges per mitotic event. While performed with three biological replicates, no mitotic events were observed with THZ1 treatment in one of the replicates. Thus, only the data from two replicates are shown in the quantitation of bridge events. For each experiment, six or more fields of cells per treatment were examined. Means are ± SEM. ∗ = *p* < 0.05 using the chi-squared test.

Micronuclei typically result from genotoxic stress (51, 52, 53, 54). The improper repair of DNA double-strand breaks (DSBs) can result in dicentric chromosomes that become trapped in anaphase bridges. As cells continue to undergo additional mitotic events, breakage–fusion–bridge events perpetuate CIN and DNA damage (52). To determine if the loss of CDK7 activity causes DSBs, the three TNBC cell lines were treated for 2 days with vehicle or THZ1 and the percentage of cells that accumulated γH2AX foci, a marker of DSBs (55), was quantified (Figs. 4, *A*–*D* and S2). While γH2AX positivity can be associated with stalled replication forks during S-phase, THZ1 treatment did not result in significant changes in S-phase cell populations (Fig. 1, *A*–*F*). These data suggest that puncta of γH2AX staining are due to DNA damage rather than an increase in cells stalled in S-phase due to replication stress. An additional CDK7 inhibitor, CT7001, was also tested in MDA-MB-231 cells (Fig. 4, *A* and *B*), as well as CRISPR/Cas9-mediated depletion of *CDK7* in MDA-MB-231 cells (Fig. 4*E*). All five models displayed significant increases in γH2AX staining following CDK7 suppression, confirming that sustained CDK7 activity is necessary to prevent genotoxic stress in the form of DSBs. To determine if the increase in DSBs was associated with chromosome bridge formation, we generated MDA-MB-231 cells that stably express eGFP-labeled Histone 2B (H2B-GFP) to visualize chromosome movement during mitosis using live-cell confocal microscopy (Fig. 5*A* and Videos S1 and S2). H2B-GFP cells were treated with 75 nM THZ1 or vehicle for 30 h and then imaged for 24 h in the continued presence of inhibitor. Consistent with our prior observations, CDK7i substantially decreased the number of mitotic events per cell, with no condensed chromosomes being observed in treated cells at this later time point (>30 h). Of the treated cells that appeared mitotic/postmitotic, the majority displayed chromatin bridges that were likely acquired in early mitoses prior to imaging. Interestingly, many of these bridges extended longer than the width of individual cells (Fig. 5, *A*–*C*). The presence of anaphase bridges suggests that the acquisition of micronuclei in response to CDK7i is due to the resolution of such bridges. Taken together, the presence of mitotic aberrations along with increased prevalence of chromatin bridges and DSBs reveals that CDK7 activity is necessary to maintain chromosome integrity and proper chromosome segregation during mitosis.

### CDK7 is necessary to maintain the expression of genes that control mitotic fidelity, including those comprising condensin

**Figure 6:**
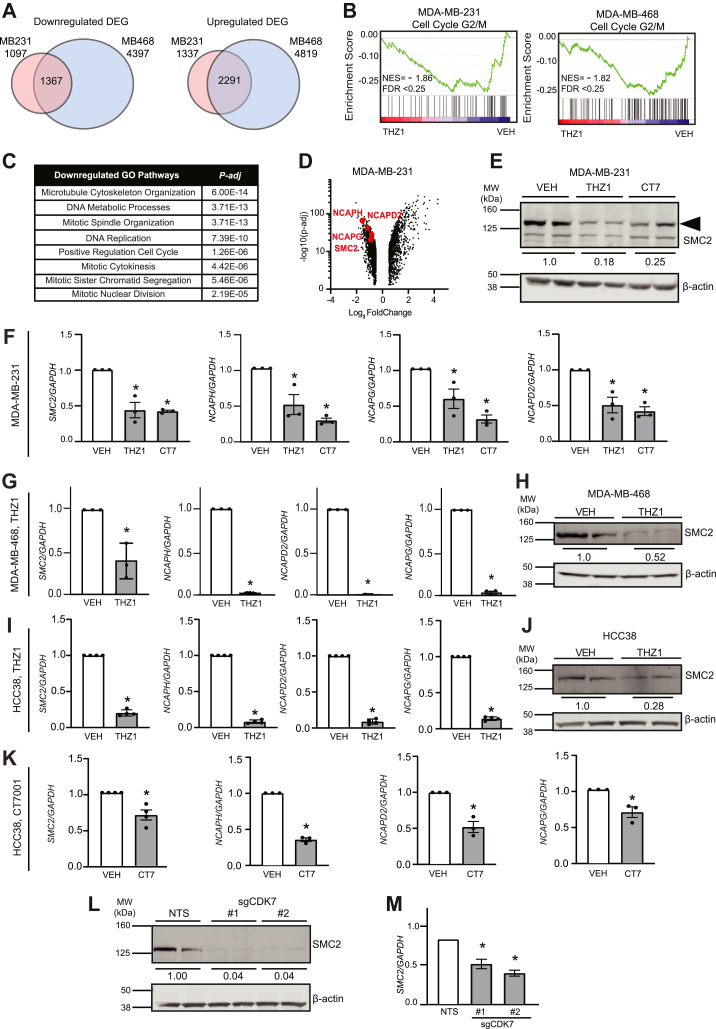
**CDK7 is necessary to maintain expression of genes that control mitotic fidelity including those comprising condensin.***A*, Venn diagram of genes that are differentially downregulated (*left*) or upregulated (*right*) in MDA-MB-231 and MDA-MB-468 cells treated with THZ1 (75 nM and 50 nM, respectively) compared with vehicle (DMSO). *B*, Gene Set Enrichment Analysis of genes in the cell cycle G2/M phase was evaluated in MDA-MB-231 (*left*) and MDA-MB-468 (*right*) cells. Normalized enrichment score (NES) and false discovery rate (FDR) are shown. *C*, table of significantly downregulated Gene Ontology pathways as assessed by evaluating the shared 1367 significantly downregulated genes in MDA-MB-231 and MDA-MB-468 cells. *D*, volcano plot of significant differentially expressed genes (DEGs) in MDA-MB-231 cells. Genes comprising the condensin complex are indicated by *red dots* with labels. *E*, representative Western blot of SMC2 protein in MDA-MB-231 cells treated with vehicle (DMSO), THZ1 (75 nM), or CT7001 (CT7, 750 nM). Values are the average of three replicates representing SMC2/β-actin. *F*, RT-PCR validation of condensin gene suppression in MDA-MB-231 cells treated as in *E*. *G* and *H*, same as *E* and *F* but using MDA-MB-468 cells treated with vehicle or THZ1 (50 nM). *I* and *J*, same as *E* and *F* but using HCC38 cells treated with vehicle or THZ1 (50 nM). *K*, RT-PCR validation of condensin gene suppression in HCC38 cells treated with CT7001 (CT7, 750 nM). *L* and *M*, MDA-MB-231 Cas9-expressing cells were transfected with nonspecific sgRNA (NTS) or sgRNA targeted to *CDK7* (sgCDK7 #1, sgCDK7 #2) for 72 h. *L*, representative blot of SMC2 protein expression following CDK7 gene disruption. Numbers represent relative SMC2/β-actin ratios. β-Actin blot is reused from Figure 3*A*. This Western was probed for both SMC2 and CDK7 (to confirm CRISPR-mediated knockout of CDK7) with β-actin as the loading control for each. M. quantitation of *SMC2* mRNA relative to *GAPDH* 72 h after transfection of MDA-MB-231 Cas9 cells with sgCDK7 *versus* nontargeting control. Bars are means ± SEM. At least three biological replicates were completed per experiment in duplicate. ∗ = *p* < 0.05.

Given the myriad of mitotic abnormalities observed following CDK7 inhibition, we used RNA-seq to identify potential mechanisms underlying these phenotypes. A total of 1367 genes were consistently downregulated and 2291 were upregulated in MDA-MB-231 and MDA-MB-468 cells treated with THZ1 compared with vehicle (Fig. 6*A*). Examination of the differentially expressed genes by gene set enrichment analysis (GSEA) (56) revealed significant (false discovery rate <0.25) suppression of genes associated with G_2_/M (Fig. 6*B*). Gene ontology (GO) of the overlapping differentially expressed genes also indicated that CDK7 loss impacts pathways associated with cell cycle progression (Fig. 6*C*). These data further confirm that sustained CDK7 activity is necessary for progressing through mitosis. We first sought to address whether this was due to MYC-driven alterations in cell cycle progression. Notably, *MYC* expression was not downregulated following THZ1 treatment in either cell line, indicating that the mitotic defects that occur in response to CDK7 disruption were independent of MYC suppression. Closer examination of repressed genes revealed that CDK7i causes a notable downregulation of numerous genes comprising the condensin (*SMC2, NCAPD2, NCAPG, NCAPG2, NCAPH, NCAPD3*) complex (Fig. 6*D* and data not shown). Condensin maintains genome stability through multiple mechanisms, including chromosome compaction, chromosome separation, enhancer looping in interphase chromosomes, and DNA repair (57, 58). In model systems, condensin loss causes chromosome missegregation, lagging chromosomes, chromosomal defects, and the formation of chromatin bridges (33, 59, 60, 61, 62), phenotypes that were also observed with the loss of CDK7 activity described above. The suppression of genes encoding several condensin subunits, including the core protein structural maintenance of chromosomes 2 (SMC2), was independently confirmed in MDA-MB-231, MDA-MB-468, and HCC38 cells using both THZ1 and CT7001 (Fig. 6, *E*–*K*). While a large number of genes were altered following CDK7i, we confirmed that the housekeeping gene, *GAPDH*, was not significantly repressed, making it an ideal internal control. These data were also recapitulated using sgRNA-directed disruption of *CDK7* in MDA-MB-231 Cas9 cells (Fig. 6, *L* and *M*). Importantly, the expression of condensin complex genes was not repressed when cells were treated with the cell cycle–selective CDK7 inhibitor, YKL-5-124, which does not directly impact transcription (Fig. S3). These results suggest that the repression of condensin complexes that occurs in response to CDK7 suppression are not simply due to alterations in cell cycle progression and the accompanying genes associated with this process. More plausible is that CDK7 directly regulates the transcription of the condensin genes. Supporting this postulate, *SMC2* expression is suppressed within 6 h of THZ1 exposure of neuroblastoma cells (63) and we found that *SMC2* expression is repressed as early as 12 h following THZ1 treatment in TNBC cells (data not shown). These rapid changes in *SMC2* mRNA levels suggest a direct effect of CDK7 on the *SMC2* gene rather than indirect effects that are a consequence of cell cycle disruption. Collectively, these data indicate that loss of CDK7 activity causes profound transcriptomic changes, including suppression of condensin complex gene expression. They also suggest that CDK7 may suppress CIN, DSBs, anaphase bridges, and mitotic catastrophe by ensuring appropriate expression of condensin to sustain chromatin and chromosome architecture.

### Silencing SMC2 phenocopies the nuclear defects induced by CDK7i

**Figure 7:**
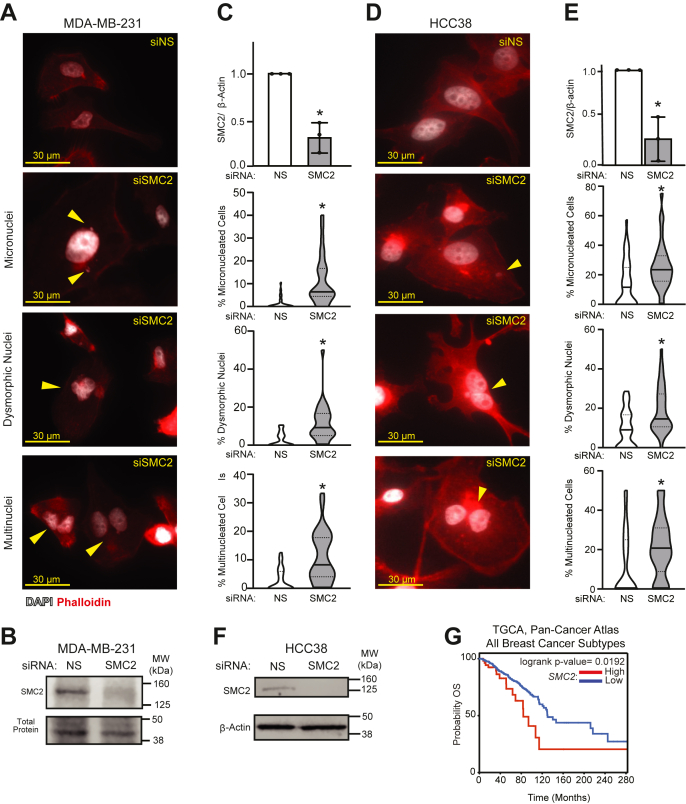
**Silencing SMC2 phenocopies the nuclear defects induced by CDK7i.***A*, seventy-two hours after siRNA silencing of *SMC2*, MDA-MB-231 cells were stained with DAPI (*white*) and phalloidin (*red*). Micronuclei, dysmorphic nuclei, and multiple nuclei are indicated by *arrowheads*. *B*, quantitation of SMC2 protein following siRNA silencing relative to total protein in MDA-MB-231 cells. *C*, quantitation of SMC2 relative to β-actin 72 h after siRNA transfection in MDA-MB-231 cells. Quantitation of nuclear phenotypes in MDA-MB-231 cells transfected with siRNA targeting *SMC2*. *D*–*F*, the same as in *A*–*C*, but in HCC38 cells. *Horizontal solid lines* are means; *dashed lines* are quartiles. Significance was evaluated by log-rank test. Three biological replicates were completed per experiment. ∗ = *p* < 0.05. *G*, overall survival (OS) curve in patients with breast cancer (all subtypes) generated with cBioPortal, stratified based on *SMC2* expression; 60 patients in the high mRNA group (μ = 11.04) and 1022 patients in the low mRNA group (μ = 10.79).

The condensin complex comprises several subunits, many of which were repressed with CDK7 blockade. While infeasible to restore individual subunits to assess their roles in mediating CDK7 function, we determined if transiently silencing the core condensin subunit, SMC2, would phenocopy the nuclear defects observed when CDK7 is inactivated (Fig. 7, *A*–*F*). Indeed, siRNA-mediated suppression of *SMC2* expression increased the percentage of cells with micronuclei, dysmorphic nuclei, or multiple nuclei, similar to inhibiting CDK7 with THZ1, CT7001, or with genetic knockout. These data indicate that suppressing condensin expression is sufficient to mimic the nuclear defects associated with CDK7 inhibition in multiple TNBC cell lines. Notably, increased *SMC2* expression is associated with poor outcomes in patients with breast cancer (Fig. 7*G*), suggesting that modulation of *SMC2* by CDK7 or other factors may have significant impact on the aggressiveness of this disease.

Inhibitors of CDK7 can disrupt SEs and reduce the expression of cell identity genes and oncogenes (18, 20). To determine if SE disruption is responsible for the loss of condensin gene expression in response to CDK7i, we used H3K27ac chromatin immunoprecipitation sequencing (ChIP-seq) to determine if SEs are associated with the condensin genes in TNBC cells. The *MYC* gene was used as a positive control for SE identification (9). Unexpectedly, the *SMC2* gene does not harbor an SE within 50 kb of the gene (Fig. S4 and data not shown). To further determine if condensin genes were associated with SEs in any cell line, we queried an SE database, dbSUPER, and confirmed that no SEs are proximally associated with the condensin genes that were regulated by THZ1 (data not shown). These data indicate that it is unlikely that proximal SE disruption is responsible for the loss of condensin gene expression and CIN that occurs in response to CDK7 inhibition.

### CDK7 is essential for chromatin compaction mediated by condensin sublooping

**Figure 8:**
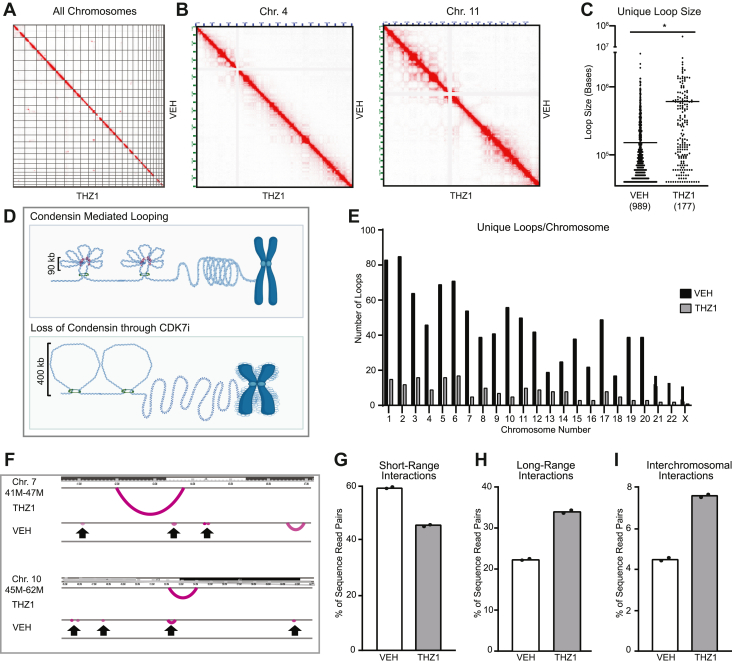
**CDK7 is essential for chromatin compaction mediated by condensin sublooping.***A*, Hi-C contact maps for all chromosomes following 48-h THZ1 treatment compared with vehicle (DMSO). *B*, Hi-C contact maps for chromosome 4 (Chr. 4) and chromosome 11 (Chr. 11). *C*, bee-swarm plot comparing loop sizes of unique loops identified in vehicle and THZ1-treated samples. Significance evaluated by Mann–Whitney U test. ∗∗∗ = *p* < 0.001. *D*, schematic of condensin mediating looping and sublooping to compact chromosomes (*top*), with the loss of sublooping in response to CDK7i suppression of condensin gene expression, resulting in misshapen, fuzzy chromosomes (*bottom*). Generated using Biorender.com. *E*, bar graph comparing the number of unique loops per chromosome in vehicle- and THZ1-treated samples. *F*, representative unique chromatin loops at chromosome 7 (Chr. 7) and chromosome 10 (Chr. 10) generated using WashU Epigenome Browser (98, 99, 100, 101). Arrows highlight small loops that are unique to vehicle-treated cells. *G*–*I*, percentage of sequence read pairs for short-range (<20 kb, *cis*), long-range (>20 kb, *cis*), and interchromosomal (*trans*) interactions in vehicle and THZ1-treated samples, respectively. n = 2.

Data presented above indicate that CDK7 is essential for ensuring the appropriate expression of several condensin-encoding genes. Given the role of condensin in maintaining chromosome architecture, including the 3D organization during mitosis, we hypothesized that CDK7 may also be essential for maintaining chromosome structure. The finding that CDK7 inhibition causes increased nuclear size (Figs. 2*D* and S2) further supports this possibility as enlarged nuclei have been correlated with alterations in chromatin compaction, loss of condensin expression, and alterations in chromosome number (48, 49, 50, 64). To directly examine the impact of CDK7 loss on chromatin folding, we used Hi-C to identify changes in 3D chromatin organization following THZ1 treatment. Unlike what has been observed for cohesin, the primary determinant of loop extrusion in interphase cells (65), CDK7 loss does not cause global changes in chromatin configuration, including no significant alterations in compartments or topologically associating domains (TADs) (Fig. 8, *A* and *B*). These data are consistent with that reported for condensin disruption in chicken DT40 cells (66). This previous study demonstrated that depletion of SMC2, and thus disruption of condensin I and II, did not impact global chromatin structure, including organization of compartments and TADs (66). As with this previous report, more subtle differences in loop size were observed with CDK7i (Fig. 8*C*). Condensins compact DNA primarily by promoting the formation of subloops that are ∼90 kb or less in size; thus a loss of condensin results in the formation of larger chromatin loops that are not subdivided (67) (Fig. 8*D*). Examination of loops that were unique to CDK7i-treated, compared with vehicle-treated, cells revealed significant alterations in chromatin loop size, as has been previously described following condensin loss in model systems (68). For unique loops, there was an ∼5-fold decrease in loops in THZ1-treated cells compared with vehicle, with an accompanying shift in mean chromatin loop size from 151 kb to 434.4 kb observed in cells treated with THZ1 compared with vehicle (Fig. 8, *C* and *E*). Examples of such shifts are shown in Figure 8*F* (69). Overall, these findings are consistent with previous studies demonstrating loss of shorter-range chromosomal interactions, resulting in increased long-range chromosomal interactions following the loss of condensin (Fig. 8, *G* and *H*) (66, 68). CDK7 inhibition also resulted in an increase in interchromosomal interactions as a proportion of unique sequence reads (THZ1 [7.61%] vs. vehicle [4.48%]) (Fig. 8*I*). In *Drosophila* BG3 cells, the loss of condensin was also associated with increased interchromosomal interactions that can contribute to translocation events in the presence of DNA damage (70, 71, 72), further supporting the role of condensin loss in mediating the effects of CDK7i on CIN in TNBC cells.

## Discussion

Herein, we report the discovery that CDK7 is a core regulator of chromosome architecture that functions, in part, by sustaining the expression of the condensin complex. Pharmacologic or genetic inhibition of CDK7 leads to pervasive mitotic dysfunction, including chromosome bridges, accumulation of DSBs, and mitotic catastrophe. Any cells that can survive these errors acquire nuclear defects such as micronuclei as well as multiple and grossly dysmorphic nuclei that inhibit further cell division. We further discovered that condensin subunit genes, in particular *SMC2*, are key downstream targets of CDK7 and that CDK7 regulates their expression in the absence of proximal SEs. The overlap in phenotypes associated with CDK7 disruption with those reported for the loss of condensin was striking, and SMC2 suppression recapitulated the nuclear phenotypes and DNA damage observed following loss of CDK7 activity. Moreover, we found that CDK7 inhibition causes shifts in DNA looping that are also consistent with a loss in condensin activity. Together, these results reveal a novel mechanism by which CDK7 maintains genome stability by ensuring sufficient condensin expression and proper chromatin folding during mitosis.

This study utilized several orthogonal tools to interrogate the functional role of CDK7 in TNBC cells, including pharmacologic and genetic approaches. Consistent disruption of mitosis with the induction of CIN was noted with several approaches, indicating that CDK7 is essential for cell growth due to its impact on mitotic progression. Moreover, the impact of CDK7 disruption on *SMC2* gene expression was consistently observed with THZ1, CT7001, and genetic knockout. A noteworthy exception is with the use of YKL-5-124, a novel inhibitor of CDK7 that disrupts its ability to phosphorylate CDK1/2, but not RNA Pol II, thus discriminating between the transcriptional and nontranscriptional effects of CDK7 (24). *SMC2* expression was not repressed by YKL-5-124, indicating that the impact of CDK7 on this gene is transcriptional and not due to indirect effects that result from disrupting cell cycle progression. Notably, SE disruption has been implicated as a key mechanism of action for the effects of CDK7i on transcription (8, 16). However, the condensin genes that are stimulated by CDK7 lack SEs as defined by H3K27ac accumulation. Thus, while genes critical for chromosome architecture and mitotic progression are dependent on CDK7 activity, our study shows that they are independent of typical SEs. As demonstrated by previous studies, many other genes that are repressed by CDK7i in TNBC do have SEs (16). Therefore, it is likely that the suppression of SE-containing genes in addition to non-SE genes, such as those encoding condensin subunits, underlie the full impact of CDK7 inhibition on genomic instability in TNBC.

Using live-cell imaging, we discovered that disrupting CDK7 leads to DSBs and the formation of long chromosomal bridges that were actively pulled between two cells (Fig. 5 and Videos S1 and S2). This indicates that sustained activity of CDK7 is necessary to prevent chromosome breakage–fusion–bridge cycles. While chromosome condensation was readily observed in vehicle-treated cells, we failed to observe condensed chromosomes following CDK7 inhibition. A similar phenomenon was previously reported in DT40 cells following *SMC2* depletion (66). Profound defects in mitotic cell fate also occurred in daughter cells that arose after an initial cell division in the presence of THZ1. Recently, Umbreit and colleagues reported that a single cell division error involving chromosome bridges can induce DNA damage, micronuclei, and chromothripsis (73), a profound rearrangement of chromosomes that increases genome complexity in cancer. Whether CDK7i and subsequent loss of *SMC2* expression also induces chromothripsis will require karyotyping or sequencing the genomes of cells treated with these inhibitors. Regardless, these data indicate that inhibiting CDK7 causes key cell division errors that could be perpetuated should any cells retain the ability to proliferate. Colony formation assays indicated that the effects of CDK7i on TNBC cells were irreversible (Fig. S1). However, if any cells escape such inhibition, we anticipate that they would have greater genomic diversity than their predecessors. Prior studies have experimentally generated cells with acquired CDK7i resistance (74, 75). It would be useful to determine if these cells have greater genomic diversity and, more importantly, if this leads to increased malignancy.

The condensin complex is necessary for the condensation of chromatin in preparation for mitosis, and decreased expression of the condensin subunits is associated with increased CIN (33, 59, 76, 77, 78, 79). As indicated above, reduced expression of condensin complex members induces similar phenotypes as observed in response to CDK7i, namely, chromatin bridges, DSBs, micronucleation, multinucleation, increased nuclear size, loss of chromatin loops, and an increase in chromatin loop size and interchromosomal interactions (33, 71, 80). The alterations in chromatin structure following CDK7i are likely limited due to the sustained expression of other major determinants of mitotic chromosome morphology, including chromokinesins such as KIF4A and KID homologues, as well as topoisomerase IIα (81, 82). Moreover, while cohesin (*SMC1 and SMC3*) gene expression was decreased in response to CDK7i, protein levels were inconsistently suppressed following CDK7 depletion or inhibition, suggesting that the impact of CDK7 on cohesin is modest. Cohesin is a major driver of chromosome organization that generates loops and TADs during interphase (83), and its loss causes profound disruption of chromatin architecture with significant loss of all loop domains (84). Since the impact of CDK7 inhibition consistently suppressed condensin subunits, but not cohesin components, alterations in chromosome structure are expected to be limited to conversion from smaller to larger loops. It is also remarkable that transcription of resident genes within the loops, as measured by RNA-seq, was not coupled to the presence of unique loops in vehicle or THZ1-treated cells. This may be in large part due to the ability of RNA Pol II and cohesin to promote interactions within genes to create small gene domains that form active chromatin compartmental domains (85). Thus, RNA Pol II or other transcription elongation complexes are stronger candidates than condensin to explain correlations between transcription and chromosome organization (85). We did note decreased phosphorylation of RNA Pol II as a result of CDK7 inhibition, suggesting that RNA Pol II changes impact condensin gene expression that then alters the formation of secondary loops, suggesting an indirect effect of CDK7 on looping.

CDK7 inhibitors are currently in clinical trials (3, 12, 13, 14, 15). Rigorous biomarkers for CDK7i response, including *CDK7* expression (31, 86) and possibly the extent of basal CIN, should facilitate patient selection if these drugs enter clinical practice. The suppression of SMC2 expression that occurs in response to CDK7i, and its ability to mediate the effect of these drugs, suggests that it may also be a useful pharmacodynamic marker. Moreover, the expression of *SMC2* was highly associated with worse patient outcomes in patients with TNBC. Extending beyond TNBC, elevated condensin gene expression is associated with poor outcomes in patients with pancreatic cancer and depletion of these genes induces CIN and apoptosis *in vitro* (79). Based on these and other published data, additional studies are warranted to determine if pretreatment expression levels of condensin complex members can predict therapeutic response to CDK7i or if changes in its expression in response to CDK7i are associated with improved patient outcomes, in TNBC as well as other aggressive forms of cancer (79, 87, 88, 89). The profound CIN observed with loss of SMC2 suggests that this protein may also be a prime therapeutic target for treating TNBC and other cancers with the same dependencies. Indeed, SMC2 has been identified as an “Achilles Gene” that is essential for pan-cancer viability (https://depmap.org/portal/) (16). SMC2 is an ATPase; thus, it may be a viable pharmacologic target for rational development of small-molecule inhibitors. Such therapies could provide a complementary approach to treat TNBC as well as other cancers where CDK7 inhibitors induce genome instability, such as the recently reported effects in SCLC and HCC (16, 24, 25, 90, 91).

In summary, the studies presented here reveal a novel mechanism of action of CDK7 involving the sustained and appropriate expression of condensin by regulating the transcription of the core subunit, *SMC2*, as well as other condensin genes. In the absence of CDK7 signaling, cells lose their normal chromosome architecture, leading to an induction of chromosomal instability and mitotic catastrophe. By ensuring proper looping during mitosis, CDK7 protects cancer cells from DNA DSBs and excessive genomic disruption as they embark on one of their most pivotal functions, cell cycle progression.

## Experimental procedures

### Cell culture and reagents

All cell lines (MDA-MB-231- RRID: CVCL_0062, HCC38- RRID: CVCL_1267, and MDA-MB-468- RRID: CVCL_0419) were purchased from American Type Culture Collection between 2015 and 2018 and maintained as directed. They were expanded, aliquoted, cryogenically stored, and used within 12 passages of thawing. Cell lines were further validated by STR mapping in 2022. MDA-MB-231, HCC38, and MDA-MB-468 cell lines were cultured in RPMI containing 10% fetal bovine serum and 1% Pen-Strep. All cells were incubated at 37 °C in 5% CO_2_. MDA-MB-231 cells that stably express Cas9 were generated using Lentiviral Cas9 Nuclease Reagents from Dharmacon (VCAS10124) according to manufacturer’s instructions. Following transduction, cells were selected using Blasticidin (Gibco #A1113903-03). MDA-MB-231(Cas9) cells were passaged in Blasticidin continuously at 20 μg/ml. Cell lines were tested for *Mycoplasma pulmonis* and *Mycoplasma* spp using MycoAlert Plus Mycoplasma Detection Kit (Lonza, LT07-703).

### Drugs and dose response curves

THZ1 (ApexBio, A8882), CT7001 hydrochloride (MedChemExpress #HY-103712A), and YKL-5-125 (MedChemExpress #HY-101257b) were resuspended in dimethyl sulfoxide (DMSO). Cells were plated in six-well plates at 100,000 cells/well and treated the following day with vehicle (DMSO) or CDK7i. Viability was assessed by trypan blue exclusion and counted on a Countess II FL Automated Cell Counter. Half maximal inhibitory concentration (IC_50_) was determined and optimal doses are as follows: MDA-MB-231, 75 nM THZ1, 750 nM CT7001, 2500 nM YKL-5-124; MDA-MB-468, 50 nM THZ1; HCC38, 50 nM THZ1, 750 nM CT7001.

### Colony formation assay

Cells were plated in six-well plates at 75,000 cells/well. At 24 h post plating, cells were treated with vehicle or CDK7i. Seventy-two hours later, cells were trypsinized and seeded in 24-well plates with drug-free, complete media. Cells were analyzed 8 days post replating with media changes every 2 days. Colonies were stained with 0.05% crystal violet, and 10% acetic acid was added to the plates before assessing absorbance at 590 nM using a Promega GloMax Explorer Plate Reader (Promega #GM3510).

### sgRNA/siRNA transfection

Disruption of the *CDK7* gene was achieved using forward transfection of sgRNA in MDA-MB-231 cells that stably express Cas9. sgRNA targeting CDK7 or a nontargeting sgRNA (sgNTS) was diluted in serum-free/Pen-Strep-free medium at a final concentration of 62.5 nM, mixed with DharmaFECT 4 Transfection Reagent at a 1:100 dilution and incubated for 20 min at 37 °C. The sgRNA/DharmaFECT mixture was added to cells and incubated for 6 h before changing to complete medium. The following sgRNAs were purchased from Dharmacon and used in this study: sgCDK7 (sgRNA #1: targeting exon 2, sgRNA #2 targeting exons 9–13; SQ-003241-01-0005) or nontargeting sgRNA #1 (U-009051-01-05). Loss of CDK7 was confirmed by Western blotting.

Transient silencing of *SMC2* was performed using forward transfection in MDA-MB-231 and HCC38 cells. siRNA targeting SMC2 or nonsilencing siRNA (siNS), targeted to firefly luciferase, was diluted in Opti-MEM medium at a final concentration of 100 nM, mixed with Lipofectamine 2000 at a 1:100 dilution, and incubated for 20 min at 37 °C. siRNA/Opti-MEM/Lipofectamine 2000 was added to cells and incubated for 6 h before changing to complete medium for the duration of the experiment. The following siRNAs were purchased from Dharmacon and used in this study: ON-TARGETplus Human SMC2 siRNA-SMARTpool (L-006836-01-0005) and siGENOME non-Targeting siRNA #2 (D-001210-02-50).

### Flow cytometry

Cells were harvested using 0.25% trypsin and fixed in 75% ethanol for 10 min at 37 °C. Fixed cells were resuspended in Propidium-Iodide/RNase A solution (100 μg/ml propidium iodide, 0.1% Nonidet P-40, 0.1% NaN3, and 1.2% RNase A) and incubated for 30 min at 37 °C. Flow cytometry was completed using the Attune NxT Flow Cytometer (Thermo Fisher). Gating was performed with FlowJo to restrict the analysis to single cells. Cell cycle analysis was completed as reported (92).

### RNA isolation and cDNA synthesis

RNA was isolated using TRIzol reagent (Ambion #155596016) followed by treatment with DNAse (DNA-free kit, Ambion #AM1906) per manufacturer’s instructions. RNA concentration was assessed using a NanoDrop One^C^ (Thermo Scientific #13-400-509). RNA quality was assessed using 1% agarose gel and examination of 28S/18S bands. Complementary DNA (cDNA) was generated using Superscript IV reverse transcriptase (Thermo Fisher #18090010) with random primers (Thermo Fisher #48190011) following manufacturer’s protocol.

### Quantitative PCR

Quantitative real-time PCR was performed on a StepOnePlus real-time PCR machine (Thermo Fisher #4376600). Gene expression was normalized to *GAPDH.* All experiments were performed at least three independent times with intraexperimental technical replicates. The following TaqMan real-time assays were purchased from Thermo Fisher: *CDK7* (Hs00361486_m1), *GAPDH* (Hs02758991_g1), *SMC2* (Hs00931422_m1), *NCAPD2* (Hs00274505_m1), *NCAPH* (Hs01010752_m1), *NCAPG* (Hs00254617_m1).

### RNA sequencing

For RNA-seq, MDA-MB-231 and MDA-MB-468 cells were treated with 75 nM and 50 nM THZ1, respectively, for 48 h (GSE160534). RNA was isolated using the RNeasy Plus Minikit (Qiagen #74007). Library preparation, sequencing, and analysis were completed by Novogene Corporation Inc using the Illumina platform with paired-end 150 bp reads mapped to hg19. Consistency across samples was tested with correlation of FPKM (fragments per kilobase of exon per million mapped fragments) values and principal component analysis. Differential expression was determined using the DESeq2 R package, where differentially expressed genes were deemed significant if the Benjamini–Hochberg adjusted *p*-value was <0.05.

### Gene set enrichment

GSEA (56) was used to assess the extent of enrichment of cell cycle signature genes obtained from MSigDB (93). GO (http://www.geneontology.org/) was used to assess biological processes that were dysregulated by THZ1. GO pathways with significantly overlapping genes were consolidated using NaviGo (94) based upon similarity score. Resnik similarity scores >1.5 indicated high levels of similarity between pathways.

### Western blots

Cells were lysed using radioimmunoprecipitation assay buffer containing sodium orthovanadate and protease (Millipore Sigma #539138) and phosphatase (PhosSTOP; Millipore Sigma #4906825001) inhibitors for 30 min on ice with intermittent vortexing. The cell lysate was spun at 10,000 RPM for 10 min at 4 °C. The protein-containing supernatant was removed and quantified by Bio-Rad Protein Assay, diluted in reducing buffer (Bio-Rad #1610737), and boiled. Protein lysates were loaded into precast polyacrylamide gels (Novex 4–20% Tris-Glycine Mini Gels, Thermo Fisher #XP00100BOX) with molecular weight markers (Licor #928-60000). Blots were transferred onto an Immobilon-FL PVDF membrane (Millipore Sigma #IPFL00010) at 0.1 Amps for 8 h. REVERT was used for total protein staining (LICOR #926-11011). All membranes were washed in Tris buffered saline with 0.05% Tween-20 (TBST) and blocked for 1 h with 5% bovine serum albumin (BSA) in TBST. The following antibodies were used for immunoblotting: anti-CDK7 (1:500), Cell Signaling Technology #2916S, RRID: AB_2077142; anti-SMC2 (1:500), Cell Signaling Technology #5394S, RRID: AB_10693943; β-Actin (1:10,000), Millipore Sigma #A2228, RRID: AB_476697; anti-total-RNA Polymerase II (1:500), Active Motif #39097, AB_2732926; anti-phospho (S2) RNA Polymerase II (1:500), Cell Signaling Technology #13499, RRID: AB_2798238. Blots were incubated in primary antibody overnight, then washed and incubated in the dark at room temperature with fluorophore-bound secondary antibodies (LICOR anti-rabbit 800CW or anti-mouse 680RD) at 1:10,000 dilutions in 5% BSA-TBST. Westerns were imaged using the LI-COR Odyssey Fc. Densitometry was performed using Image Studio (LICOR), and relative protein levels were quantified in relation to β-Actin or total protein.

### Live-cell imaging, incucyte

An Incucyte Zoom (Essen BioScience) was used to image MDA-MB-231, MDA-MB-468, and HCC38 cells treated with vehicle or THZ1 (75 nM, 50 nM, and 50 nM, respectively) over 4 days as described (41). Images were collected at 20× magnification every 15 min on phase. Fields were divided into nine equal regions. One cell/field was followed through mitosis; the timing required for transition through mitosis and the fate of it and its daughter cells were assessed. The beginning of mitosis was identified upon visualization of the metaphase plate with a subsequent step back in the imaging start time by 10 min. The end of mitosis is established when two daughter cells were observed to readhere to the plate. Mitotic outcomes were binned into the following categories: exit and die (obvious death of the cell), exit and divide (a subsequent mitotic event), die in mitosis (obvious cell death prior to cell readherence to the plate), prolonged interphase (time spent without dividing again), and failed cytokinesis (a cell begins mitosis, does not form two daughter cells, but remains alive).

### Nuclear phenotyping

Nuclear morphology was evaluated after 48 h of treatment with vehicle or CDK7i or after 72 h transient siRNA transfection or sgRNA transfection. Cells were fixed with 4% formaldehyde in PBS, permeabilized with 0.1% Triton X-100 in PBS, and blocked with 5% BSA in PBS. F-actin was stained with Texas Red-X phalloidin (Invitrogen, T7471), and nuclei were counterstained with ProLong Diamond Mountant with DAPI (Invitrogen #P36962). Cells were imaged at 20×, 40×, or 60× magnification using a Leica DMS200 microscope or Keyence BZ-X810.

Quantitation of nuclear contour irregularities was achieved using elliptical Fourier analysis. Raw images of DAPI-stained nuclei were manually separated into individual JPEG files containing one nucleus each. These images were then evaluated using a custom R code (utilizing the Momocs package) (95) that extracted outlines of each nucleus and generated elliptical Fourier approximations of the nuclear contours using 15 elliptical harmonics. Nuclear contour irregularities were calculated as EFC ratios by comparing the sum of the sizes of elliptical Fourier harmonics 2 to 15 to the size of the first harmonic. EFC ratios from a pool of nuclei that were visually determined to be at the threshold of being considered dysmorphic were calculated, and the average EFC ratio of this pool was used as a threshold value for the entire analysis; any nucleus that yielded an EFC ratio that was less than or equal to this threshold was labeled dysmorphic.

Nuclear size was evaluated using ImageJ. DAPI images were converted to grayscale to transform the pixel’s color information into a brightness measurement. The area of each nucleus was measured using the measure pixels function under the analysis tool in ImageJ.

DNA damage was assessed after 48 h treatment with vehicle or CDK7i or 72 h after transient siRNA transfection or sgRNA transfection. γH2AX staining utilized an antibody conjugated to Alexa Fluor 488 at a 1:50 dilution (Abcam #AB195188, RRID: AB_1645352). Cells were imaged at 20×, 40×, or 60× magnification using a Leica DMS200 microscope or Keyence BZ-X810. Cells were deemed γH2AX positive if they contained ≥5 foci/nucleus.

### Confocal imaging

To visualize chromatin, MDA-MB-231 cells were transfected with pEGFPN1-H2B (Addgene, #11680) and selected with 700 μg/ml Geneticin for 14 days. Cells were then sorted based on GFP intensity with cells of medium intensity being expanded. Pooled cells were then treated with either DMSO or 75 nM THZ1. After 30 h, they were imaged for an additional 20 to 24 h (37 °C, 95% humidity, 5% CO2) by a Leica SP8 confocal microscope. At 40X magnification, 5 to 10 fields were imaged/treatment. Time lapse avi files were generated using Leica LAS X software (Leica Microsystems, GmbH), converted into m4v video files, reviewed, and quantified.

### High-throughput chromosome conformation capture

MDA-MB-231 cells were treated with vehicle or THZ1 for 48 h in duplicate (GSE223785). Cells were washed twice with PBS, trypsinized, and pelleted. About 7 to 10 × 10^6^ cells were cross-linked with 2% formaldehyde. Frozen samples were sent to Arima Genomics. Cross-linked chromatin was digested using a proprietary restriction enzyme cocktail and then labeled with biotinylated nucleotide. Spatially proximal digested ends of DNA were ligated, purified, fragmented using Bioruptor Pico from Diagenode, and enriched using Streptavidin beads. Enriched fragments were subjected to a custom library protocol utilizing Swift Biosciences Accel-NGS 2S Plus DNA Library Kit (Cat # 26148, 26248, 26396, 26596, 26696, 26796, 26896, or 269384) to produce Arima-HiC libraries for sequencing. Libraries were sequenced using an Illumina NovaSeq 6000 to generate 150 bp paired-end reads, with a read depth of ∼537 to 781M reads/sample. The resulting Arima-HiC data were analyzed with Juicer v1.6 (96) for A/B compartments, TADs, and chromatin loop calls, which were visualized using Juicebox (97). Chromatin loops were further visualized using WashU Epigenome Browser (98, 99, 100, 101).

### Chromatin immunoprecipitation sequencing

Using a previously published method for histone 3 lysine 27 acetylated (H3K27ac) ChIP-seq in MDA-MB-231 cells (41), we identified SEs in MDA-MB-231 (GSE95222) and MDA-MB-468 (GSE160534) cells using H3K27ac Rank Ordering Super-Enhancer software (ROSE) (17, 102). Gene-associated SEs were identified if they were within 50 kilobases (kb) of the gene.

### Public database analysis

Kaplan–Meier curves for TCGA Pan-Cancer data were generated using cBioPortal to assess microarray data of 1082 patients with breast cancer (all subtypes); patients were stratified based on the expression of *SMC2* with 60 patients in the high mRNA group (μ = 11.04) and 1022 patients in the low mRNA group (μ = 10.79). Patients were included regardless of treatment history.

Using dbSUPER (https://asntech.org/dbsuper/index.php), we determined the presence of SEs within 50 kb of selected gene loci (103).

### Statistical analyses

Statistical significance was determined using two-tailed Student’s *t* test (*in vitro* assays), log-rank test (Kaplan–Meier Curves), Wilcoxon signed-rank test (nuclear size analysis), and Mann–Whitney U test (Hi-C loop size) with *p*-values <0.05 being considered statistically significant. All *in vitro* experiments were performed at least three independent times, each in duplicate or triplicate technical replicates. The mean of the biological replicates is shown with variability indicated by standard error of the mean. The mean of technical replicates is shown with variability indicated by standard deviation.

## Data availability

Raw data from RNA-seq (MDA-MB-231 and MDA-MB-468) and ChIP-seq (MDA-MB-468) were deposited in GEO, under accession number GSE160534. Raw and processed data from the Hi-C experiment were deposited in GEO, under accession number GSE223785.

## Supporting information

This article contains supporting information.

## Conflict of interest

The authors declare that they have no conflicts of interest with the contents of this article.
