# Supplementary figures and images for: Disruption of CDK7 signaling leads to catastrophic chromosomal instability coupled with a loss of condensin-mediated chromatin compaction
Source: J Biol Chem. 2023 May 17;299(7):104834. doi: 10.1016/j.jbc.2023.104834 (PMC10300262; doi:10.1016/j.jbc.2023.104834)

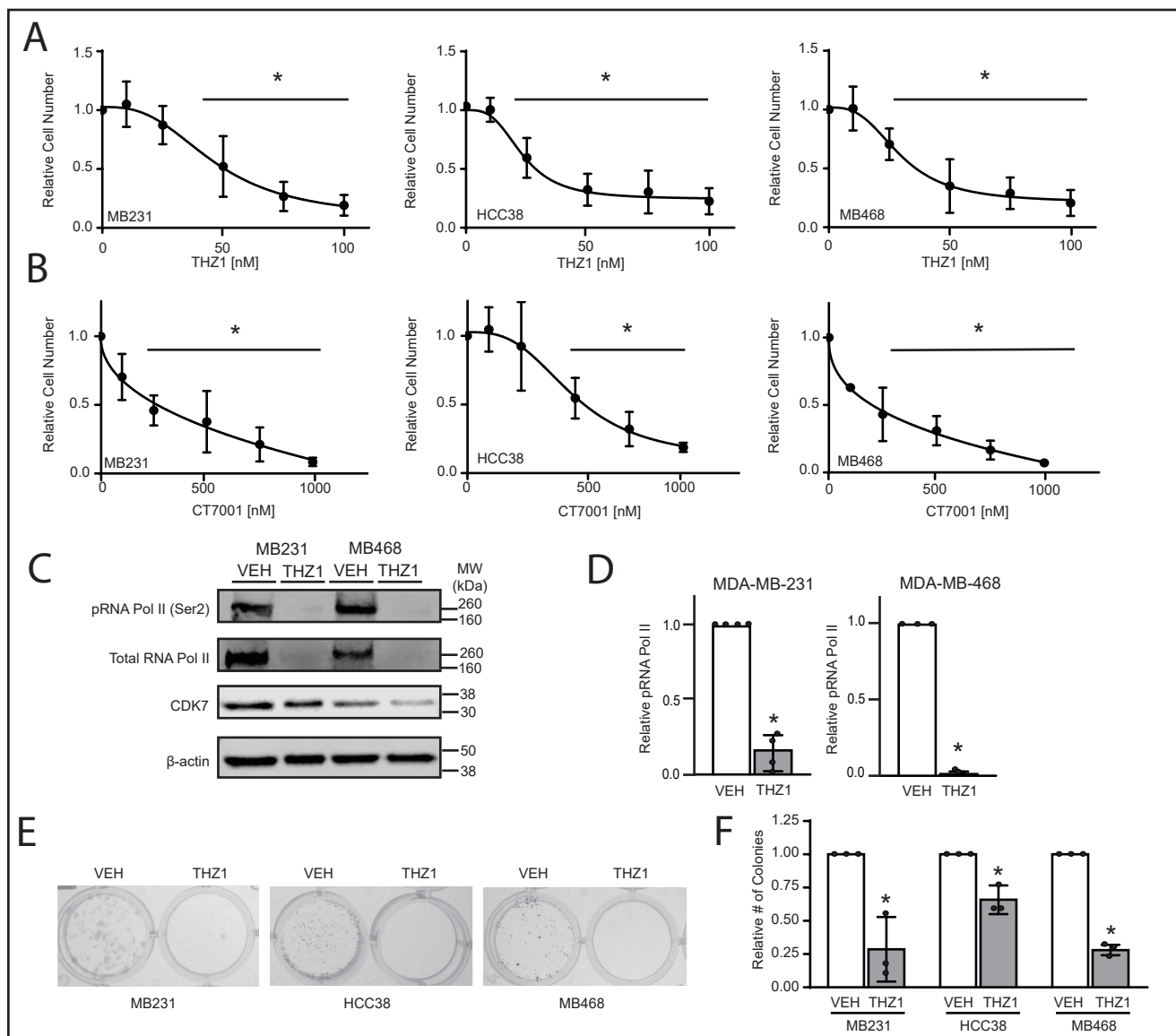

Supplemental Figure 1

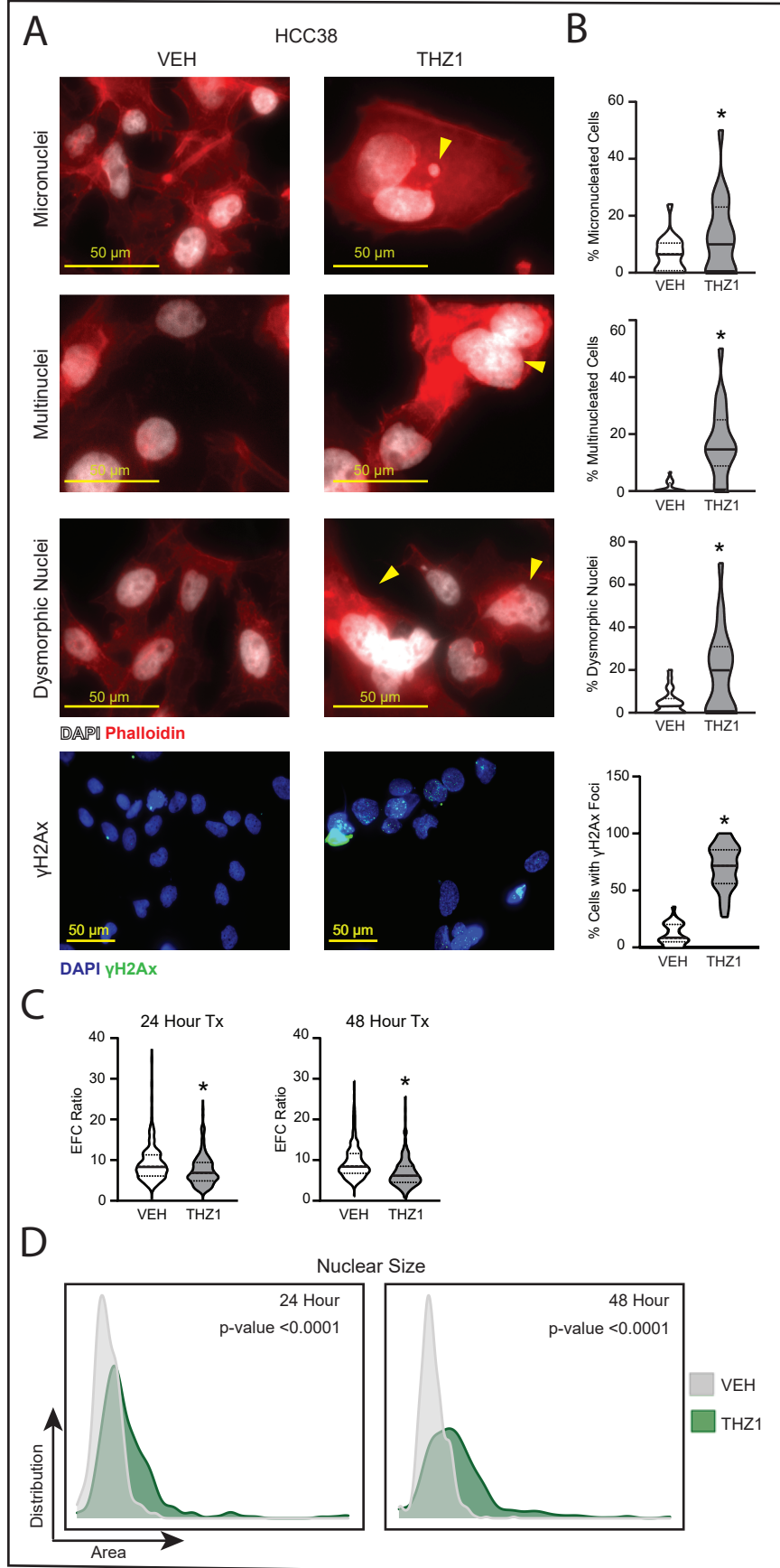

Supplemental Figure 2

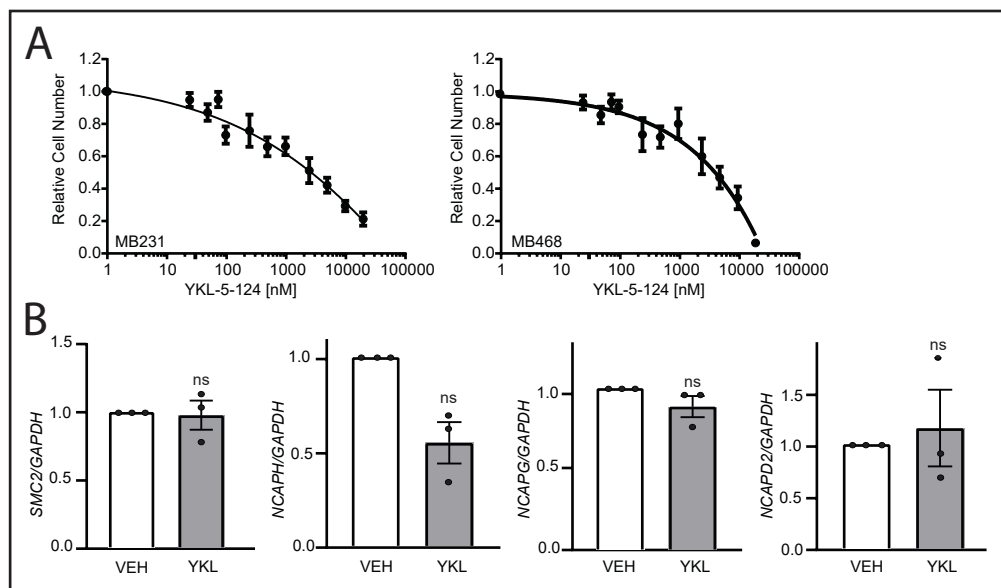

Supplemental Figure 3

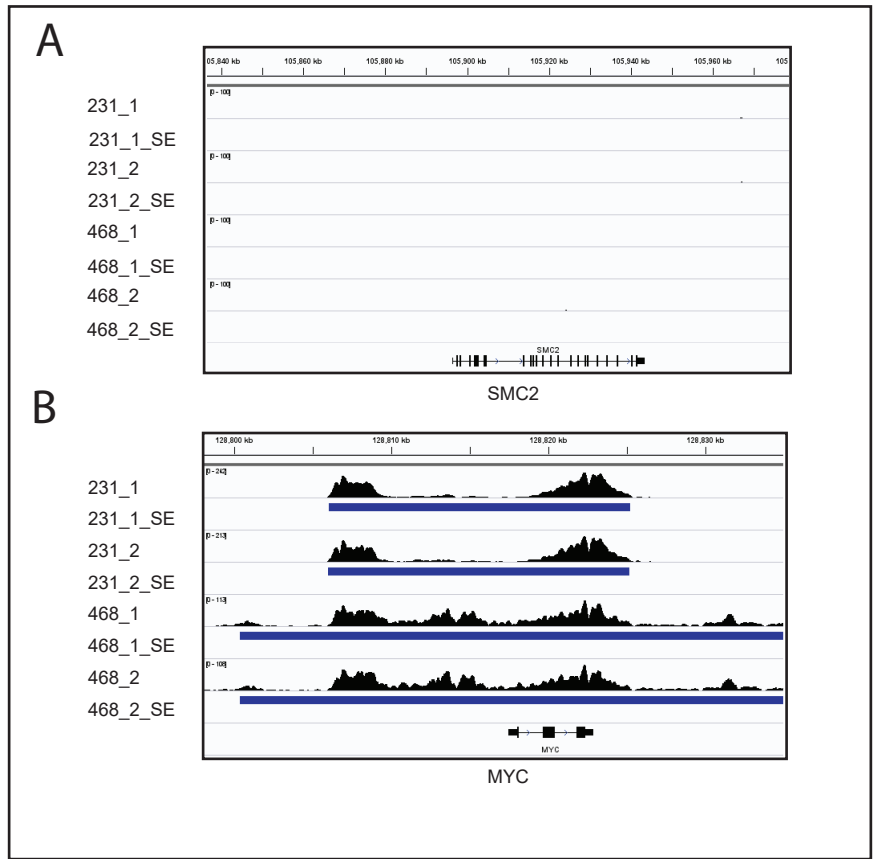

Supplemental Figure 4

Supplement: Supporting information [file mmc3.pdf]
