# Supplementary material for: Disruption of CDK7 signaling leads to catastrophic chromosomal instability coupled with a loss of condensin-mediated chromatin compaction
Source: J Biol Chem. 2023 May 17;299(7):104834. doi: 10.1016/j.jbc.2023.104834 (PMC10300262; doi:10.1016/j.jbc.2023.104834)
Supplement: Supporting information Figure Legends [file mmc4.docx]

Supporting Information Figure Legends

Supporting Figure S1. Sustained CDK7 activity is necessary to maintain TNBC cell growth. A. MDA-MB-231, HCC38, and MDA-MB-468 cells were treated with increasing doses of the CDK7 inhibitor, THZ1, for 72 hr. Changes in cell number were quantified using crystal violet staining. B. Same as A, but with increasing concentrations of the CDK7 inhibitor, CT7001. C. MDA-MB-231 and MDA-MB-468 cells were treated with THZ1 (75 nM or 50 nM, respectively) for 48 hr. Western blots were probed for phosphorylated RNA Pol II at serine 2 or the C terminal domain, total RNA Pol II, and CDK7. A representative western blot is shown. D. Quantitation of activated/phosphorylated RNA Pol II relative to β-actin in MDA-MB-231 and MDA-MB-468 cells after 48 hr treatment with THZ1. E. Colony formation assays using cells pre-treated with vehicle (DMSO) or THZ1 (231: 75 nM; 468 and HCC38: 50 nM) for 72 hr. Cells were then re-plated and analyzed after 8 days of growth in drug-free complete media. F. Quantitation of colonies. * = *p*<0.05. n=3-4 experiments, each completed in triplicate. Values are means ± SD.

Supporting Figure S2. CDK7 inhibition induces nuclear hallmarks of chromosome instability in HCC38 cells. A. HCC38 cells were treated with vehicle (DMSO) or THZ1 (50 nM) for 72 hr. Cells were stained with DAPI (nuclei, white) and phalloidin (actin, red). Micronuclei, dysmorphic nuclei, and multiple nuclei are indicated by arrowheads. B. Quantitation of the percentage of cells with each phenotype. Horizontal solid lines represent means, dashed lines are quartiles. At least 600 cells were counted/treatment/cell line. C. Elliptical Fourier coefficient (EFC) ratios of HCC38 nuclei following 24 and 48-hr treatment with THZ1. D. Nuclear size of HCC38 nuclei following 24 and 48-hr treatment with THZ1. Three independent biological replicates were completed/experiment. Wilcoxon signed-rank test was used to assess statistical significance of nuclear size changes. * = *p*<0.05.

Supporting Figure S3. The cell cycle selective CDK7 inhibitor, YKL-5-124, does not suppress SMC2 expression. A. MDA-MB-231 and MDA-MB-468 cells were treated for 72 hr with increasing concentrations of the cell cycle selective CDK7 inhibitor YKL-5-124 and cell number assessed. B. Quantitation of *SMC2*, *NCAPH*, *NCAPD2,* and *NCAPG* mRNA relative to *GAPDH* 72 hr after YKL-5-124 treatment of MDA-MB-231 cells. Values shown are means ± SD for three independent experiments. Bars are means ± SEM. *ns= not significant.*

Supporting Figure S4. *SMC2* lacks super-enhancers. Duplicate H3K27ac ChIP-seq tracks for MDA-MB-231 (231_1 and 231_2) and MDA-MB-468 (468_1 and 468_2) cells. Super-enhancers were identified by the Rank Ordering of Super-Enhancers (ROSE) and the lack of such enhancers is shown in duplicates (*e.g.,* 231_1_SE). *MYC* has a well-documented super-enhancer [9, 103] that is confirmed here in our ChIP-seq (blue line) as a positive control.
